# Exploring the first delay: a qualitative study of home deliveries in Makwanpur district Nepal

**DOI:** 10.1186/1471-2393-14-89

**Published:** 2014-02-27

**Authors:** Joanna Morrison, Rita Thapa, Machhindra Basnet, Bharat Budhathoki, Kirti Tumbahangphe, Dharma Manandhar, Anthony Costello, David Osrin

**Affiliations:** 1 Institute of Global Health, University College London, 30 Guilford Street, London WC1N 1EH, UK; 2 MIRA, PO Box 921, Thapathali, Kathmandu, Nepal

**Keywords:** Formative, Context, Maternal, Newborn, Delay

## Abstract

**Background:**

In many low-income countries women tend to deliver at home, and delays in receiving appropriate maternal care can be fatal. A contextual understanding of these delays is important if countries are to meet development targets for maternal health. We present qualitative research with women who delivered at home in rural Nepal, to gain a contemporary understanding of the context where we are testing the effectiveness of an intervention to increase institutional deliveries.

**Methods:**

We purposively sampled women who had recently delivered at home and interviewed them to explore their reasons for home delivery. Interviews were recorded, transcribed and analysed using thematic content analysis. We used the ‘delays’ model discussed in the literature to frame our analysis.

**Results:**

Usually a combination of factors prevented women from delivering in health institutions. Many women were aware of the benefits of institutional delivery yet their status in the home restricted their access to health facilities. Often they did not wish to bring shame on their family by going against their wishes, or through showing their body in a health institution. They often felt unable to demand the organisation of transportation because this may cause financial problems for their family. Some felt that government incentives were insufficient. Often, a lack of family support at the time of delivery meant that women delivered at home. Past bad experience, and poor quality health services, also prevented women from having an institutional delivery.

**Conclusions:**

Formative research is important to develop an understanding of local context. Sociocultural issues, perceived accessibility of health services, and perceived quality of care were all important barriers preventing institutional delivery. Targeting one factor alone may not be effective in increasing institutional deliveries. Our intervention encourages communities to develop local responses to address the factors preventing institutional delivery through women’s groups and improved health facility management. We will monitor perceptions of health services over time to help us understand the effectiveness of the intervention.

## Background

Progress is being made towards the Millennium Development Goal (MDG) of reducing maternal mortality by 75% by the year 2015. Yet an estimated 287,000 women still die from complications in pregnancy or childbirth every year [1]. Globally, newborn mortality has been slow to respond to MDG efforts, and recent analysis suggests that 41% of deaths in children under five happen during the first 28 days of life [2]. Increasing access to good quality institutional care during labour and delivery has been identified as a key strategy in increasing maternal and infant survival. Delays in reaching a health facility, and delays in receiving the correct care in the right place, at the right time, can be fatal [3]. In countries like Nepal, where 63% of women deliver at home [4], there is work to be done to reduce the first delay - the delay in deciding to seek care. In their review of the literature, Thaddeus and Maine found that sociocultural issues, perceived accessibility, and perceived quality of care were the main factors contributing to the delay in deciding to seek care. In order to reduce delays there is a need to design and test interventions that address both demand and supply side issues [5].

### Demand and supply issues in Nepal

Over the past few years the Government of Nepal has initiated policies to increase access to maternal health care. In 2009 a national policy of free institutional delivery care, and cash transfers for women delivering in health institutions, was initiated (Aama Surakshya Programme) [6]. This programme of support has largely been successfully implemented [7] and it is likely that the programme has contributed to increases in institutional deliveries over recent years [8]. The 2011 National Demographic and Health Survey (NDHS) found that only 5% of women delivering at home did so because of the costs of institutional care [4]. The Government of Nepal is also trying to address human resource challenges in providing maternal and newborn care, for example through establishing targets for enrolling health workers in the Skilled Birth Attendant training course. It is also encouraging periphery level health facilities to provide 24 hour delivery services, beyond the normal opening hours of 10 am to 2 pm. These Government policies demonstrate commitment to address the barriers to institutional delivery, and indicate that the policy environment may be receptive to other low cost interventions that may reduce delays in care seeking.

It is in this context that our research partnership between the Institute of Global Health, University College London, and MIRA Nepal is testing the effectiveness of a demand and supply side intervention. We are using a cluster randomised controlled trial design to test the effect of community mobilisation through women’s groups, along with health management committee (HFMC) strengthening, on institutional delivery and delivery with a trained health worker [9]. Our intervention seeks to support behaviour change within communities and within health facilities through a participatory planning and action approach [10]. In three-day workshops, we support 21 HFMCs and their communities in intervention clusters to assess their health facility and plan actions to improve health services. We provide on-going monitoring and help to establish organisational networks. Also in intervention areas, 203 monthly women’s groups are facilitated by female community health volunteers to discuss the barriers to institutional delivery, and address these with their community.

Yet behaviour change is difficult to initiate and sustain. There is evidence to suggest that changes in home care practices are more likely to occur if interventions build upon existing practices, identify and target the most receptive community members, bolster local skills and priorities, recognise constraints on human agency and feature mobilisation of the community [11-14]. Formative research is beneficial in enabling interventions to meet these criteria. An understanding of context helps to develop appropriate interventions, and estimate the potential for behaviour change [15,16]. We present findings from formative qualitative research that was conducted to help us explore how our intervention might be effective, and plan our process evaluation data collection. Specifically we sought to understand the reasons for home deliveries, and explore how the Aama Programme affected women who delivered at home.

## Methods

### Setting

Makwanpur district is south-west of Kathmandu and has a population of 420,477 [17], of whom most live in rural areas and are engaged in agriculture. It has a mid-range human development index (0.47) [18]. The Tamangs, of Tibeto-Burman origin, are the largest ethnic group. There are gender imbalances in literacy: 54% of women are literate, compared with 73% of men [19]. The government district hospital has 50 beds, and there are two private hospitals, of which one has facilities for conducting deliveries. There is one health facility in each of 43 Village Development Committee subdivisions of the district, and four Primary Health Centres (PHCs), ten Health Posts (HPs), and 30 Sub Health Posts (SHPs). We conducted a survey of all 43 HFMCs in 2009, and found that only 15 had participated in an orientation programme about their roles and responsibilities, and 18 had initiated more than four activities in the past year [9]. At baseline, we found that one Doctor, four Staff Nurses, and three Auxiliary Nurse Midwives had received skilled birth attendant training [20].

We have been supporting community mobilisation through women’s groups in the district since 2001, and from 1^st^ November 2001 to 31^st^ October 2003, 24 Village Development Committee clusters participated in a cluster randomised controlled trial testing the impact of women’s groups on neonatal mortality [21]. There was a 30% decrease in mortality in intervention areas, and there is a continuing secular trend of improved newborn survival in the district. Yet the number of deliveries in health institutions remains low. Baseline data (collected from November 2009 to September 2010) show that 28% of deliveries occurred in government health institutions, and a trained health worker attended 30% of deliveries. Traditional Birth Attendant utilisation is low in the district, and mothers-in-law usually attend deliveries [22].

### Data collection

We conducted data collection during the first 6 months of the trial. We purposively sampled from six clusters receiving the women’s group and HFMC intervention and six control clusters, with equal numbers of Primary Health Centres, Health Posts, and Sub Health Posts in each type of cluster. We sampled from intervention and control areas because we sought to explore baseline contextual differences that may be important to understand the effect of the intervention. We conducted semi-structured interviews with three women per cluster who had delivered at home in the preceding six months. None of these women had had a trained health worker or a traditional birth attendant at their delivery. Women were located using our trial surveillance system and were approached in their homes. Most interviews were conducted separately from family members. We only sampled women who had had a normal delivery, as we did not wish to further disturb families who had experienced a neonatal death and had already given detailed information about it to our surveillance team. Data were collected in Nepali by Nepalese researchers who were experience in conducting qualitative research and were trained in qualitative methods by the Nepali speaking Principle Investigator (JM). The Principle Investigator accompanied researchers in the field, to observe initial data collection. Topic guides were piloted and adjusted in an iterative way. Piloted data were also analysed. We took verbal consent to participate and discussions were recorded and transcribed in Nepali. Qualitative data loses some meaning when it is translated, therefore we analysed half of the transcripts in Nepali, and half of the transcripts in English. The Principal Investigator does not read Nepalese and therefore translation of some data was required to analyse the data together with Nepalese researchers. Researchers who had collected data and the Principle Investigator manually analysed English and Nepali qualitative data together using thematic content analysis [23]. We read transcripts, discussed emergent themes, and coded transcripts according to them using data from two study clusters. We discussed and refined themes and used this coding structure for the remainder of the transcripts.

This study was granted ethical approval by the University College London Ethics Committee, and the Nepal Health Research Council.

## Results

We collected data from 33 women who had delivered at home. We could not analyse three interviews because recordings were of bad quality. For eight of these women this was their first delivery. 22 women were of Tamang ethnicity, seven were from disadvantaged ethnic groups (Rai and Chepang), one was Brahmin, one was Newar, and ethnicity data were missing for two women. We asked women to explain the main reason for delivering at home, but very few were able to do so. Usually, a combination of factors influenced the decision to have a home delivery. Using the framework developed by Thaddeus and Maine, we listed the number of women who stayed at home due to socioeconomic and cultural factors; perceived accessibility; and perceived quality of care (Table 1).

**Table 1:** Factors contributing to the first delay

***Contributing factors***	***Number of women***	***Thaddeus and Maine classification***
Bad experience with health services, and/or bad perception of health services	12	Perceived quality of care
Embarrassment	12	Socioeconomic and cultural
Lack of family support	10	Socioeconomic and cultural
Means of reaching health facility (including arranging men to carry)	8	Perceived accessibility
Money	8	Socioeconomic and cultural
Short labour	4	Perceived accessibility
Labour occurred at night	4	Cultural
Distance to health facility	2	Perceived accessibility
Use of Shaman	2	Cultural
Culture of home delivery	1	Cultural

### Socioeconomic and cultural factors

#### Illness factors

Recognition of the need for institutional delivery is influenced by concepts of what is normal, what constitutes a problem or a risk, and problem causation. Care seeking in Nepal often involves consultation with a range of health care providers, and research has shown that traditional healers, or shamans, are often the first source of treatment [14,24,25]. These may be Dhami Jhankri’s, family members or neighbours who are believed to have healing abilities [14]. In our study we sampled women who had normal deliveries and we did not explore care seeking during illness in any depth. Most women said that if they experienced a problem during labour they would go to a health facility: “I thought if difficulties occurred then I would go to hospital. But my baby was born at home”. Only a few women from intervention and control areas mentioned that they called the shaman to the delivery, or that they call the shaman when they have health problems. Where a family member was a shaman it was more difficult to have an institutional delivery. “My grandfather-in-law is a shaman. He said that I could give birth at home. I told my husband that doctors had told me to come to the health post and that they would treat me well. My husband said, ‘grandfather has told you that you will give birth very soon so you don’t need to go to the health post’”.

A few women said that it was more convenient to deliver at home, and that it was unusual to deliver in a health facility “we have no culture to go other places during delivery…no-one has gone to hospital for delivery from here”. When some women suggested delivering in a health facility, their families felt it was unnecessary.

#### Women’s status

The status of women is a cross-cutting theme in every barrier to institutional delivery, and affects women of all ethnicities, in intervention and control clusters. In Nepal, women usually move to their husband’s household after marriage, and often have very little say in household decision-making [26,27]. There is social pressure to behave in support of their marital home, and women often prioritised the needs of the family before their needs: “What would we discuss? My family said that as far as possible I should deliver the baby at home, and if it’s not possible, then they could take me to hospital. My mother-in-law said that delivering the baby at home is good because it is difficult to take clothes from our home to the hospital”. The pressure not to bring shame on the family sometimes prevented them from informing others about their pregnancy, expressing their discomfort or signs of illness, or letting health workers physically examine them: “I felt shy as I thought that the nurses would look everywhere”. “My reputation is worth more than the 1000 rupees (incentive)”. Their low status also affected access to economic resources to be able to seek care independently if necessary: “I didn’t have money, so I didn’t go to hospital”. Their dependence on the family also affected their ability to act without family support: “he said to me, ‘you don’t need to go (to the health post)… money will be spent… I told him, ‘you don’t need to worry about money, after I am well I will work hard and would repay the money’… he didn’t say anything, he just bowed his head”.

We found that a lack of awareness about the benefits of institutional delivery did not prevent women from delivering in a health institution. Women from intervention and control areas who delivered at home were generally aware that it could be better for their health and the health of their child to deliver in a health institution: “It is good to deliver your baby in the hospital. They take good care (of you)”.

### Perceived accessibility

#### Distance and transportation

Both distance and transportation were contributing factors in the decision to deliver at home in all types of clusters. Most of the women in our sample lived in hilly areas, and even those living relatively close to a health facility had difficulty during labour because paths were narrow and difficult to navigate. Several women gave birth at night and were scared to leave their homes: “I had planned to deliver at the health facility but I had labour pain at night and the health facility is far from here… the path is very difficult, we could go immediately if the road was like it is in cities”. When women delivered during a festival or at a time when male household members were not there, it was difficult to find enough people to carry them, and this prevented them from going to a health facility. “I was planning to go, but no one was there to take me to the health post”. For some, the uncertainty of time of birth prevented organising this in advance, but a few women reported that there was no possibility for them to go to a health institution as their labour was short: “It was not my wish to deliver the baby at home. The labour pain was short… I was alone so that I was not able to go to hospital. Nobody was there to take me”. A few women reported that they delivered at home while transport was being arranged. Families often supported a plan for institutional delivery during pregnancy, but for some women support waned at the time of delivery – either because the supportive family member was not at home, or because they had changed their mind.

#### Cost

Linked to concerns about accessibility of the health facility were concerns about the cost of food and accommodation for accompanying family members. All those reporting concerns about cost were of Tamang or Rai ethnicity, from all types of health facility catchment area, but mainly in control areas. When they needed men to carry them to a health facility, women also worried about these costs: “I couldn’t go by myself to the health post and I needed men to carry me. If we used men to carry me then we should feed them and give them money. So thinking, ‘why should we spend money?’ I decided to deliver my baby at home”.

Most women knew about the free delivery care and delivery incentive. Only a few had not heard about the incentive, or had heard incorrect information about their eligibility. Most thought it was a good idea and enabled the repayment of loans, buying medicine, and buying food. For those who delivered at home due to money concerns, it was not sufficient to cover all the expenses they could incur. In a few clusters, women had heard that money was not given immediately, but most felt the discussion of the incentive was irrelevant to them as they were not eligible and had not received it.

### Perceived quality of care

Half of the women who reported bad experiences with the health services spoke about Health Posts, mostly from participants in intervention areas. Most of the women in our study had antenatal care during their pregnancy, and were encouraged to deliver in the health institution by health workers. Women were discouraged from delivering in health institutions when they or their family members had had negative interactions during previous treatment or antenatal care: “When I went there they said, ‘We are not experienced so we don’t know whether you are pregnant or not.’ Then after that I didn’t go anymore”.

Some women didn’t go to health facilities when they were in labour because they thought they would be closed: “I would have been taken to the hospital if it was open, but there was no-one there”. One woman had gone for a previous delivery and was almost turned away because it was after 2 pm. Some were scared of delivering on the way, and this happened to one woman who returned home when the health facility was closed. Three women who delivered at home had gone to the health facility during labour and were told to return when their labour had progressed further.

Another factor that discouraged women from delivering in a health facility was their lack of freedom to move around, eat and drink what they wished, and have their family there to support them: “At home we can move around as we like and hold onto anything we like but in hospital they make us stay on the bed and they do what they like. If I deliver at home my family members hold and support me and that makes it easier for me to have the baby”. They were also restricted in that they had to purchase whatever food is available: “At home we can eat whenever we like, but in hospital we cannot. At home we can arrange milk for the baby, and we can care for the baby”. Their lack of freedom to deliver in the position of their choice was also related to concerns about privacy. In health institutions, women were usually made to deliver lying on their backs, on a delivery bed, often with their feet in stirrups. “We can give birth at home by sitting, but in hospital we need to be lying down… everybody comes and sees, so it’s not good to go (to the health institution)”. In this position it is much more difficult to maintain your own modesty than if you are free to move around.

## Discussion

Usually, a combination of factors prevented women from having an institutional delivery. Economic barriers may have affected women in control areas more, and perceived quality of care in health posts may have affected women in intervention areas more – although these barriers usually interact with other factors and our sample was small. Women were unlikely to deliver their babies in a far away health facility, when it was dark and late and there was no one to carry them. If there is a chance that the health facility might be closed, they are even more unlikely to attempt the journey while in labour. If a woman’s privacy is not maintained, and she is not treated with respect, she won’t go to a health institution. The familial expectation that women can and should deliver at home exists in this context, and women and families were concerned about incurring costs that were often considered avoidable and unnecessary. Although the incentive was encouraging institutional deliveries, some study participants, felt that the incentive alone was not enough, and improvements in quality of care were also necessary.

### Limitations

Our study has shown that the role of family members in increasing institutional deliveries is key, and our study would have been strengthened if we had collected data from this respondent group. We have tried to overcome this limitation by collecting data from men and women, and HFMCs in a concurrent study about quality of care in the same study areas [28].

We only interviewed women who had had a normal delivery at home. If a delivery becomes complicated, the reasons for delays in the home may change. For example, the cost of delivery may become more of a concern if families feel that a complicated delivery is more costly than a normal delivery. Several national studies have focussed on care-seeking during complications, and if the goal is to create a national norm of institutional delivery it is also important to consider women having normal deliveries.

### Aama programme

Many women were concerned about the costs of institutional deliveries, despite being aware of the Aama programme. A recent rapid assessment of the Aama programme also found high levels of awareness about the programme, but found that 23% of women were still paying some of the costs of their delivery [29]. Another study in Makwanpur District reported that the incentive was perceived to be insufficient to fully cover delivery expenses, was not always received on time [28]. To ensure proper management for the Aama programme, monitoring is necessary, and could be addressed by HFMCs within our intervention.

### Quality of care

Our study indicates that perceived quality of care is one of the main issues that causes delays in the home. Although distance and transportation are also barriers to institutional delivery [30], there is some evidence that quality is more important than distance to health facilities [31]. Women in our study reported on the importance of good staff behaviour, privacy, and the availability of staff. These issues were given less importance by HFMCs and communities members in a concurrent study [28]. If communities are unaware of the key issues that discourage women from delivering in health institutions, these issues will not be the focus of advocacy efforts. It is important that HFMC and community members actively seek the opinions of women of reproductive age to understand what affects women’s motivation to deliver in health institutions.

The aspects of quality that were discussed are amenable to change and could be targeted in our intervention to positively affect utilisation. We aim to stimulate a dialogue between women, HFMCs and communities, although we acknowledge that changes in attitudes and institutional cultures may take time [32]. A recent study to improve quality of care in hospitals in Malawi suggests that staff stability, leadership, training, and ongoing support from district staff and external coaches trained in quality improvement methods are necessary to sustain the process [33]. Generally, there is little evidence regarding how quality improvement initiatives can improve outcomes [34-36]. It is important to understand how our intervention is or is not effective, and we will monitor quality of care as it could be an important driver of reduction in the first delay.

It has been suggested that a lack of awareness and education among women prevents institutional deliveries [28,37]. Literacy rates and educational achievement of women lag behind that of men, with women in the plains being particularly disadvantaged [17]. Most of the women in our sample belonged to a Buddhist indigenous minority group, who are said to enjoy more freedom within the private sphere, and better access to and control over household resources than other Hindu women [38,39]. Yet in the public sphere these women are often excluded from participation due to caste, language or religious dominance of other groups [27]. We found that despite their low educational attainment and social exclusion, many women in our study were aware that institutional delivery could be good for their health and their baby’s health. It appears that health promotion efforts and advice from health workers at antenatal care visits may be increasing awareness. We found that women’s status in the home, and the extent of family support, were more significant determinants of home delivery than awareness and education [40], despite their relative ethnic autonomy in the home. Although some research supports these findings [40], high profile studies like the NDHS [4] and the Maternal Mortality and Morbidity study in 2008 [41] may be misleading in their indication that family support is not a major barrier to institutional delivery. It is important to capture the complexity of factors affecting place of delivery, including the fact that women often put the desires of others before their own needs [26]. The literature from the region also indicates that enduring childbirth without complaining or disturbing others is a source of pride [42-44] and if any fuss is made it may be viewed as shameful and uncivilised [45].

Our study indicates that knowledge about the health benefits of institutional delivery may not be sufficient to change behaviour. Women also need to be able to communicate that they are in labour, that they want to go to a health institution, and their family needs to be ready to respond immediately. Through our intervention we are targeting women, families, and communities, as we believe that this may be more successful than targeting women alone.

## Conclusion

Understanding and preventing delays is essential to improve maternal health and survival. In our study we focused on the first delay and found that the Thaddeus and Maine model still applies in our context. Our intervention takes a participatory approach and is designed to address local issues defined by communities. We have found that perceived quality of care – including better management of the Aama programme - should be improved, and that family support is a key issue causing delays at home. We will monitor these issues in intervention and control areas in order to understand how the intervention is or is not effective.

## Competing interests

The authors declare that they have no competing interests.

## Authors’ contributions

JM designed the study, analysed data and wrote the first draft of the manuscript. RT and MB collected and analysed data. BB and KT contributed to study design and data management. DM, AC and DO conceived of the study and advised in the interpretation of data. All authors read and approved the final manuscript.

## Pre-publication history

The pre-publication history for this paper can be accessed here:

http://www.biomedcentral.com/1471-2393/14/89/prepub
